# Construction and evaluation of the knowledge graph and large model question-answering system for Jin San Zhen therapy: a tool study for primary care and general practice

**DOI:** 10.3389/fmed.2026.1755583

**Published:** 2026-04-09

**Authors:** Junjie Chen, Minting Luo, Jianchao Chen, Guangming Luo, Genxin Li, Chubin Lei, Dongjing Chen, Jin Yu, Kunru Gu

**Affiliations:** 1 School of Acupuncture and Rehabilitation Clinical Medicine, Guangzhou University of Chinese Medicine, Guangzhou, Guangdong, China; 2 Panyu Hospital of Traditional Chinese Medicine, Guangzhou, Guangdong, China; 3 Australian Chinese Medicine Commerce Association, Sydney, NSW, Australia; 4 Guangzhou Zihetang Traditional Chinese Medicine Co., Ltd., Guangzhou, Guangdong, China

**Keywords:** intelligent question answering, Jin San Zhen, knowledge graph, large language model, traditional Chinese medicine

## Abstract

**Background:**

Jin San Zhen acupuncture therapy is a classical Traditional Chinese Medicine (TCM) school originating from the Lingnan region of China. It is widely used in China for central nervous system diseases, internal medical conditions, and various pain disorders, benefiting a large number of patients. However, the related clinical evidence and expert experience are scattered across journal articles and monographs, without systematic curation or structured presentation, making it difficult for frontline clinicians and trainees to access in a timely manner. Although general-purpose large language models (LLMs) can generate answers, they are prone to “hallucinations” and lack traceable evidence-based support.

**Objective:**

Based on Chinese clinical research literature and authoritative monographs from the past decade, this study aimed to construct a Knowledge Graph (KG) for Jin San Zhen and to develop an intelligent question–answering (QA) system that combines the KG with LLMs to answer clinical and educational questions related to Jin San Zhen.

**Methods:**

We searched Chinese databases such as China National Knowledge Infrastructure (CNKI), Wanfang, and CQVIP for clinical studies published between 2016 and 2025 in which Jin San Zhen was the main intervention, and incorporated information on point combinations and clinical practice from four authoritative monographs. Following a PRISMA-style selection process (905 initial records → 416 after deduplication → 191 included studies) we designed an ontology comprising seven entity types (diseases, acupoints, acupoint combinations, treatment plans, etc.) and nine relation types. We used the Qwen3-MAX LLM for information extraction, supplemented by manual verification, and ultimately constructed the KG in Neo4j. We evaluated the KG intrinsically using Precision, Recall, and F1 metrics against a human-annotated gold standard derived from stratified sampling (*n* = 149 treatment plans from *N* = 298, 95% confidence level, 5% margin of error), with inter-annotator agreement assessed on 81 overlapping annotations. We then designed a retrieval-augmented generation (RAG) workflow, in which user queries are parsed by an LLM into a limited set of query types, Cipher templates are used to retrieve the graph, structured records are returned, and the LLM generates natural language answers that can be traced back to the original literature. We described the scale and characteristics of the KG using node and relation statistics, and developed 60 evaluation questions covering common query types and major disease categories. Two TCM acupuncture experts were invited to rate, under a double-blind design, the answers produced by three systems—a “KG+LLM template model,” a “KG+LLM hybrid model” incorporating fuzzy entity matching and enhanced retrieval strategies, and an “LLM-only model”—on three dimensions (correctness, professionalism, and completeness) using a 1–5 scale. Paired *t*-tests were used to compare differences across all pairwise model combinations.

**Results:**

The final KG contained 921 nodes and 3,745 relations, including more than 80 diseases, over 360 standardized acupoints, 55 core acupoint combinations, and 298 treatment plans, systematically representing the “disease–plan–acupoint” relationships and efficacy characteristics of Jin San Zhen. Intrinsic evaluation showed that the KG achieved post-refinement F1 scores of 0.952 for main acupoints (*P* = 0.959, *R* = 0.949) and 0.859 for auxiliary acupoints (*P* = 0.984, *R* = 0.858), with inter-annotator F1 of 0.991 and 0.999, respectively. Across 60 evaluation questions, the KG ± LLM hybrid model achieved the highest mean scores on all three dimensions (correctness: 5.00; professionalism: 5.00; completeness: 4.40), significantly outperforming both the KG ± LLM template model (4.75, 4.77, 4.03) and the LLM-only model (4.05, 3.65, 4.12; all pairwise comparisons *p* < 0.01). Notably, the hybrid model resolved the completeness limitation observed in the template-based approach, while both KG-enhanced systems produced answers fully traceable to source literature across all 60 questions, with no fabricated claims detected by expert reviewers. Expert feedback indicated that the hybrid model's layered presentation—distinguishing high-confidence graph-derived content from supplementary general knowledge—provides particularly strong clinical reference value.

**Conclusion:**

Compared with a general-purpose LLM, the Jin San Zhen knowledge-graph–based QA system—particularly with the tiered confidence generation strategy—markedly improves the accuracy, professionalism, and completeness of answers while providing traceable evidence with explicit confidence labeling. The system thus has the potential to serve as an auxiliary tool for primary care and general practitioners to rapidly access information on Jin San Zhen, perform evidence integration, and support teaching. Future prospective studies in real-world clinical settings are needed to evaluate its actual impact on decision quality and patient outcomes.

## 1 Introduction

Jin San Zhen is a highly influential specialized acupuncture system in contemporary Chinese acupuncture, created and continuously refined since the 1960 s by Professor Jin Rui, a renowned Lingnan acupuncturist at Guangzhou University of Chinese Medicine (1). In the history of acupuncture development in China, Jin San Zhen is not only an important representative of the Lingnan acupuncture school but also a key achievement integrating inheritance and innovation in modern acupuncture. Its name derives from its core therapeutic concept of “three-point combinations,” in which three key acupoints are carefully selected for different conditions to form relatively fixed prescriptions, such as “Temporal Three Needles” for post-stroke sequelae (2), “Intelligence Three Needles” for intellectual disability and dementia (3), and “Four Spirit Needles” for cognitive impairment (4). This standardized prescription strategy, characterized by a minimal yet essential selection of points, has enabled a shift from traditional experience-based acupuncture toward a more replicable, teachable, and researchable standardized approach. Jin San Zhen was included among the first 64 nationally recognized TCM academic schools in China. Its academic value is reflected in three main aspects: it establishes a systematic mapping between diseases and acupoints/acupoint combinations, providing a clear framework for “searching points by disease” and standardized clinical teaching; it formulates a complete set of operational procedures, including specific needling techniques, insertion depth, and manipulation essentials, which confer high reproducibility in clinical practice; and it actively incorporates modern medical theories to reinterpret traditional acupuncture from the perspectives of neuroanatomy and pathophysiology, thereby promoting the modernization and internationalization of acupuncture.

In clinical practice, Jin San Zhen has attracted wide attention due to its definite efficacy, broad indications, and standardized operations. Its applications primarily focus on three areas. First, central and peripheral nervous system diseases such as cerebral palsy in children, post-stroke sequelae (5), and Parkinson's disease (6); in particular, multiple domestic studies have reported significant improvements in the rehabilitation of pediatric cerebral palsy (7). Second, mental, psychological, and sleep-related disorders (8), including depression, anxiety, and insomnia, for which prescriptions such as “Calming Spirit Needles” are representative and have distinctive clinical efficacy in calming the mind, alleviating anxiety, and improving sleep. Third, musculoskeletal and chronic pain conditions such as frozen shoulder (9), lumbar disc herniation (10), and osteoarthritis (11), where, based on local point selection and meridian theory, Jin San Zhen often achieves stable improvements in pain relief and functional recovery. In addition, Jin San Zhen has shown unique advantages in allergic rhinitis, chronic gastrointestinal functional disorders, and certain gynecologic conditions. A large body of clinical reports suggests that Jin San Zhen is characterized by relatively rapid onset, sustained therapeutic effects, few adverse reactions, and high safety. At present, Jin San Zhen has expanded from its origins in the Lingnan region to nationwide adoption, with more than a thousand academic papers and dozens of monographs published (12–14), and hundreds of medical institutions across China routinely providing Jin San Zhen treatment. Internationally, the therapy has spread to more than 30 countries and regions, including the United States, the United Kingdom, Canada, Australia, and Malaysia, becoming an important component of international exchange and education in TCM acupuncture. Several disciples of the Jin school have opened Jin San Zhen clinics overseas, enabling patients from diverse cultural backgrounds to benefit from this therapy. Nevertheless, knowledge related to Jin San Zhen has long been dispersed across journal articles, conference proceedings, textbooks, and monographs, with inconsistent terminology for diseases, acupoints, prescriptions, and operational details. This has led to severe knowledge fragmentation and a lack of structured organization. Frontline clinicians—particularly those in primary care and general practice—often find it difficult to obtain systematic, reliable, and traceable evidence-based information on Jin San Zhen in a timely manner, which to some extent constrains its standardized dissemination and inheritance on a broader scale.

With the rapid development of artificial intelligence, LLMs (15) are increasingly applied in the medical field. In recent years, large models such as GPT-4, Qwen, and DeepSeek (16) have been used for medical QA (17), automated summarization of electronic health records, rapid retrieval and summarization of scientific literature, diagnostic prediction, and virtual health consultation for the public (18). Compared with traditional rule-based and retrieval-based systems, LLMs exhibit clear advantages in natural language understanding and generation, and can better comprehend complex medical questions and produce relatively structured responses. In the field of TCM (19), the combination of LLMs and Knowledge Graphs (KG) has been used for the digitization and knowledge mining of classical texts (20), intelligent syndrome differentiation assistance, TCM formula generation and optimization, and mapping between Chinese and Western medical terminologies (21), demonstrating strong capabilities in cross-text information integration. However, general-purpose LLMs heavily depend on their pretraining corpora, and their internal “knowledge base” lacks explicit timestamps and source annotations. In specialized domains where evidence is fragmented and rapidly evolving, they often exhibit “hallucinations,” generating seemingly plausible but actually incorrect content in the absence of reliable support. For a characteristic acupuncture system such as Jin San Zhen, which combines traditional experience with modern clinical research, relying solely on general-purpose LLMs for QA makes it difficult to ensure the accuracy, professionalism, and traceability of answers, and may mislead clinical decision-making and education.

Given this situation, there is currently a lack of a systematic KG specifically for Jin San Zhen therapy and an integrated intelligent QA tool oriented toward clinical practice and education. While RAG-based QA frameworks have been reported in general medicine and TCM domains, our primary contribution lies in domain-specific knowledge modeling and curation for Jin San Zhen therapy, rather than proposing novel AI architectures. Specifically, we address the unique challenges of: 1. Standardizing heterogeneous terminology for acupoints, combinations, and disease indications across scattered literature sources; 2. Designing a clinically meaningful ontology that captures the “disease–plan–acupoint” prescription logic specific to Jin San Zhen; 3. Establishing a curated, evidence-based knowledge resource that can serve as a replicable model for other specialized TCM therapies. In this study, we integrated clinical research literature from Chinese databases between 2016 and 2025 in which Jin San Zhen was the main intervention, together with the core content of the authoritative monographs *Jin Rui's Academic Thoughts and Integrated Experience of Jin San Zhen Therapy* (12), *Comprehensive Clinical Experience Atlas of Jin San Zhen Therapy* (13), *Essentials of Jin San Zhen Therapy*, and *Atlas of Jin San Zhen Point Combinations* (14). Our objectives were: 1. To construct a specialized KG for Jin San Zhen therapy; 2. To develop a retrieval-augmented QA system that combines the KG with an LLM; 3. To compare, through expert evaluation, the answer quality of a “knowledge-graph–enhanced LLM” vs. a “general LLM.” We aim to provide a reusable digital pathway for evidence integration, standardized inheritance, and rational application of Jin San Zhen in primary care and general practice.

## 2 Materials and methods

KG (22) provide a feasible solution to the aforementioned problems. By designing an appropriate ontology in advance and modeling core elements such as “diseases,” “standard acupoints,” “Jin San Zhen acupoint combinations,” “specific treatment plans,” “treatment courses and operational details,” and “efficacy indicators and conclusions” as nodes, and linking them with relations such as “disease–plan,” “plan–main/accompanying acupoints,” and “disease–high-frequency acupoints,” unstructured textual information originally scattered across literature and monographs can be transformed into a structured and computable “Jin San Zhen knowledge network.” In the Jin San Zhen context, this multilayered “disease–acupoint–acupoint combination–plan–efficacy” network not only helps clarify the internal logic of point selection in the Jin school and unify terminology across different sources, but also provides an authoritative and controllable external knowledge source for LLM-based QA. Before answering a specific clinical question, the LLM can first retrieve relevant structured information from the KG and then generate natural language responses based on it, thereby substantially mitigating hallucinations and enabling answers whose content can be traced back to the literature.

**Figure 1:**
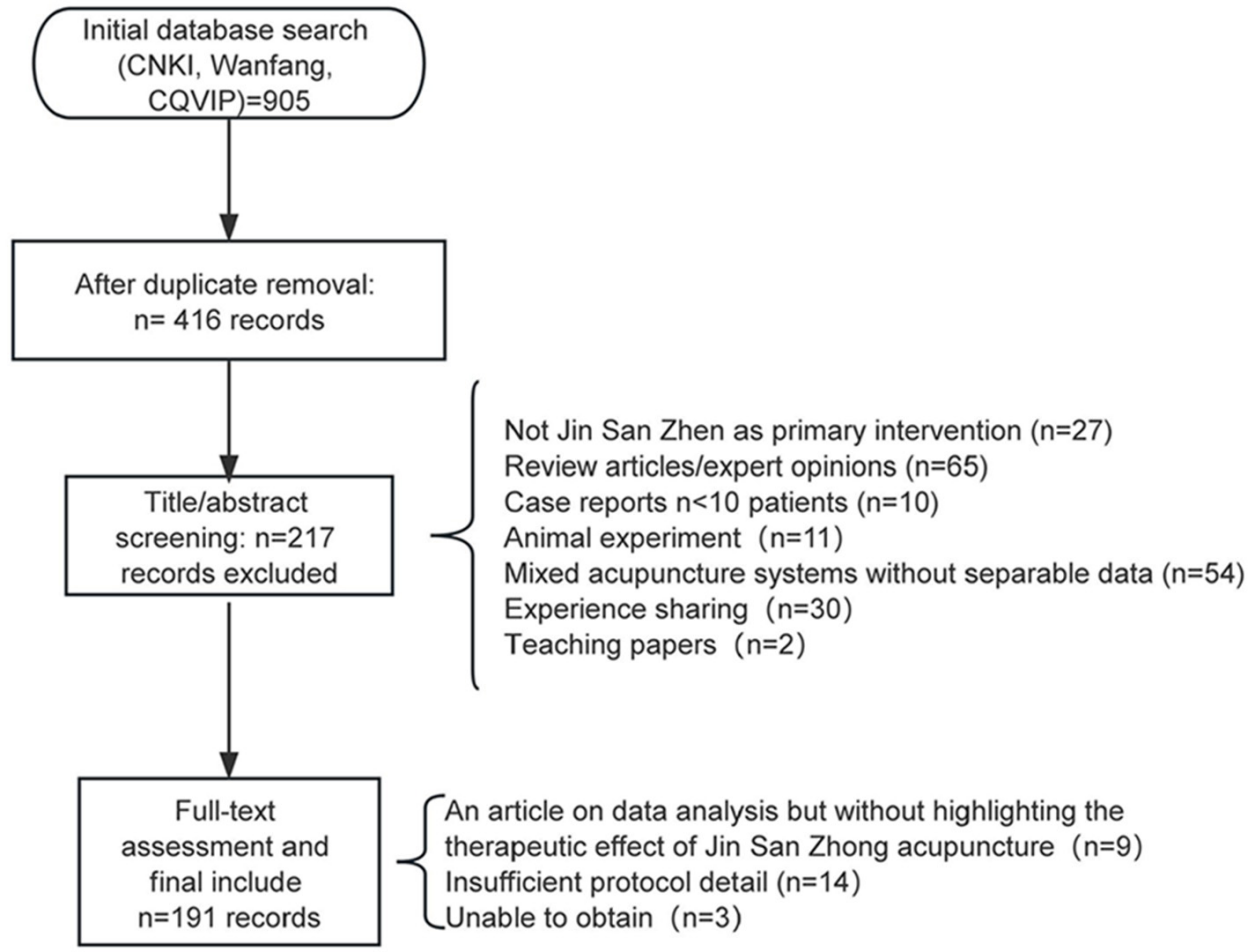
Literature screening process.

**Figure 2:**
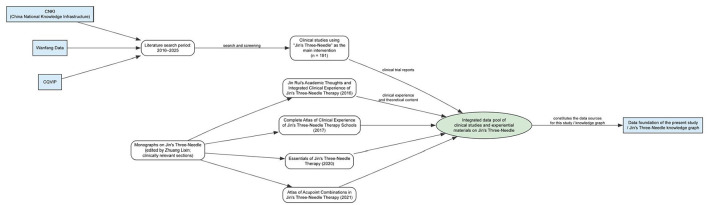
Literature and monograph screening chart.

Based on this background, we used Jin San Zhen therapy as the case study to construct a systematic KG and, on this basis, to develop an LLM-based QA system using retrieval-augmented generation (RAG) (23) technology. We searched CNKI, Wanfang, and CQVIP. The 191 included clinical studies were selected through systematic database searching with explicit inclusion criteria: published 2016–2025 in Chinese peer-reviewed journals, Jin San Zhen as primary intervention (not merely adjunctive), explicit treatment protocol description (acupoints, needling methods, course), reported clinical outcomes (efficacy rates, symptom scores, or objective measures), and clearly defined study population. Exclusion criteria included small case reports (*n* < 10 patients), review articles or expert opinions without original clinical data, studies mixing multiple acupuncture systems without separable Jin San Zhen data, and duplicate publications of the same patient cohort. Of the 905 initial records identified across the *three* databases, 489 duplicates were removed, leaving 416 unique records. During title and abstract screening, 199 records were excluded for the following reasons: Jin San Zhen was not the primary intervention (*n* = 27); review articles or expert opinions without original clinical data (*n* = 65); case reports with fewer than 10 patients (*n* = 10); animal experiments (*n* = 11); mixed acupuncture systems without separable Jin San Zhen data (*n* = 54); experience-sharing articles without systematic outcome reporting (*n* = 30); and teaching-focused papers (*n* = 2). The remaining 217 articles underwent full-text assessment, of which 26 were further excluded: articles focused on data analysis without highlighting the therapeutic effect of Jin San Zhen (*n* = 9); insufficient protocol detail to extract treatment plan information (*n* = 14); and inability to obtain the full text (*n* = 3). This process yielded 191 studies for final inclusion (Figure 1). We included studies with varying methodological quality ranging from randomized controlled trials to observational case series to comprehensively represent the existing evidence base rather than selectively including only high-quality studies, ensuring the KG reflects the full spectrum of published Jin San Zhen literature while users can assess study design and quality through the detailed information provided in each treatment plan record including sample size, control group presence, and outcome measures used. Higher-quality studies, particularly those reporting total effective rates exceeding 80% with statistical significance vs. control groups, were assigned higher effect_level values in the KG, enabling users to prioritize evidence when multiple treatment plans exist for the same condition. The 191 included studies, together with the monograph content described below, served a dual purpose: they constituted the primary data source for constructing the KG, and the KG itself was subjected to intrinsic evaluation using Precision, Recall, and F1 metrics (described in the KG Intrinsic Evaluation subsection below). Thus, the included studies are integral to both the knowledge base construction and its quantitative quality assessment. At the same time, we systematically incorporated clinically relevant sections from four monographs authored by Jin San Zhen lineage successor Zhuang Lixing—Jin Rui's Academic Thoughts and Integrated Experience of Jin San Zhen Therapy (2016), Comprehensive Clinical Experience Atlas of Jin San Zhen Therapy (2017), Essentials of Jin San Zhen Therapy (2020), and *Atlas of Jin San Zhen Point Combinations* (2021)—as shown in Figure 2, along with the 46 core three-needle prescriptions summarized in the section on prescription composition, such as Nasal Three Needles, Eye Three Needles, Stomach Three Needles, Contracture Three Needles, Hand Three Needles, and Foot Three Needles, for which standardized descriptions of location, function, indications, and operational specifications are provided. The two source types served complementary and mutually corroborating roles: the 191 clinical studies provided disease-specific treatment protocols, acupoint usage patterns, and efficacy evidence, while the *four* monographs supplied authoritative definitions of the 46 core combinations with standardized compositions, needling methods, and classical indications. Clinical practice validated monograph-defined prescriptions, and monograph standards in turn guided the normalization of heterogeneous clinical descriptions. These literature sources and monograph materials constituted the primary data sources for constructing the KG in this study.

For ontology design of the KG (24), we centered on the clinically most relevant “disease–treatment plan–acupoint/acupoint combination” chain and defined six core entity types (25): standardized acupoint (Acupoint), Jin San Zhen acupoint combination (AcupointCombo), disease (Disease), treatment plan (TreatmentPlan), local point within a combination (ComboLocalPoint, referring to non-standard local stimulation sites), and raw point name (RawPointName, preserving the original point expressions in the literature). The selection of six entity types and eight relationship types was determined through iterative consultation with three senior Jin San Zhen practitioners (Yu Jin, Chen Jianchao, and Luo Guangming) and preliminary analysis of representative literature, aiming to balance clinical relevance, query expressiveness, and construction feasibility. These entity and relationship types were validated to adequately represent the core clinical knowledge patterns observed in Jin San Zhen practice, including disease indications, standardized acupoint prescriptions, treatment protocols, and efficacy evidence. The primary clinical query patterns we aimed to support include identifying which treatment plans exist for a specific disease, determining the core acupoint combinations in a given plan, assessing the evidence for treating a particular condition, and discovering which diseases can be addressed by a specific acupoint combination. Our pilot construction with initial articles confirmed that this schema could represent the extracted clinical information without excessive complexity that would hinder graph construction and maintenance.

Given the heterogeneity of efficacy reporting in source literature, we adopted a two-tier representation in the TreatmentPlan entity where the effect_text attribute preserves the original authors' verbatim descriptions (e.g., “The overall effective rate is 89.5%.” or “significant improvement in Barthel Index, *p* < 0.01”), enabling users to trace back to the original language, while the effect_level attribute provides a standardized categorical classification to facilitate comparison. High efficacy (Level 2) was assigned when the total effective rate reached or exceeded 80%, or when explicit terms like “marked effectiveness” or “significant efficacy” appeared, or when statistically significant superiority with large effect sizes was reported. Moderate efficacy (Level 1) was assigned when the total effective rate ranged from 60%−79%, or when terms like “effective” or “improvement” appeared, or when moderate effect sizes were documented. Low or unclear efficacy (Level 0) was assigned when the rate fell below 60%, when descriptions were ambiguous, or when differences were non-significant. This classification was applied consistently by the research team during manual verification of LLM-extracted records to ensure internal consistency across the knowledge base.

**Figure 3:**
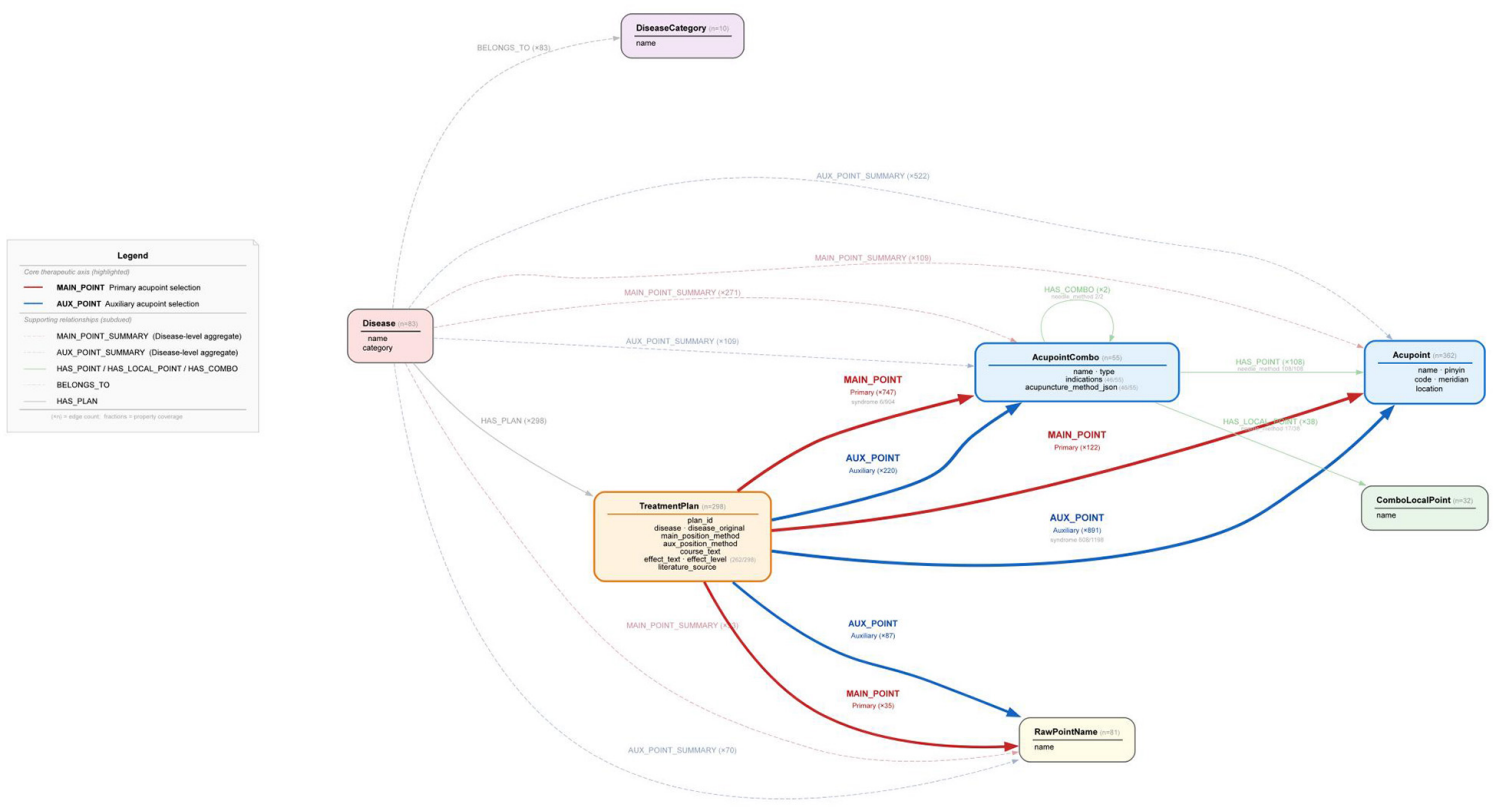
Entity and relationship diagram.

Information extraction combined LLM processing with manual verification where all LLM-generated entity and relation records underwent human review to correct extraction errors, resolve ambiguities in acupoint naming, and verify accurate mapping to standardized codes. Common correction patterns included distinguishing between main and auxiliary acupoints, standardizing variant acupoint names to GBT codes, and clarifying ambiguous efficacy descriptions. This hybrid approach ensured the KG faithfully represents the source literature while maintaining internal consistency. The Acupoint entity records information such as acupoint name, international standard code, pinyin, meridian, and body surface location. The AcupointCombo entity records the combination name, subtype (e.g., “Temporal 1 Needle,” “Temporal 2 Needle”), needling method for each acupoint, and diseases primarily treated by the combination. The TreatmentPlan entity is uniquely identified by a plan_id, linked to the corresponding disease, and includes detailed fields such as treatment method description, course description, efficacy description, textual description of treatment site, efficacy level, and whether electroacupuncture, moxibustion, or pharmacotherapy are combined. This design transforms scattered disease names, acupoint names, three-needle combinations, and operational measures across different literature sources into unified, searchable structured data. Entities are further connected by multiple relation types, including HAS_PLAN relations from diseases to treatment plans, MAIN_POINT and AUX_POINT relations from plans to primary and auxiliary acupoints or combinations, HAS_POINT and HAS_LOCAL_POINT relations from acupoint combinations to standardized acupoints or local points, and MAIN_POINT_SUMMARY and AUX_POINT_SUMMARY relations that summarize high-frequency main and auxiliary acupoints across multiple plans for a given disease, this relational network, illustrated in Figure 3, directly supports typical clinical questions such as “Which Jin San Zhen plans are commonly used for a given disease?,” “What are the core three needles and associated points in a given plan?,” “Which main and auxiliary points are most frequently used in treating a specific condition?,” and “Which acupoints constitute a given Jin San Zhen combination, and how should each be needled?.”

For information extraction, we adopted a strategy of “LLM-based extraction with manual verification” (26). We selected Qwen3-MAX as the information extraction engine based on several considerations including its strong performance on Chinese medical texts as documented in recent benchmarking studies, its large context window (256K tokens) enabling processing of lengthy clinical articles, its reliable structured output generation in JSON format, and its stable API access through Alibaba Cloud DashScope. Alternative models we considered, such as GPT-4, ChatGLM-Pro, and smaller open-source models, either had limitations in Chinese medical terminology comprehension, smaller context windows, or less stable API availability. While we did not conduct formal inter-annotator agreement measurements for the extraction step, manual verification of the LLM-extracted records revealed recurring error patterns that required correction. The LLM occasionally misclassified acupoints as “main” when the source text indicated them as “auxiliary” or vice versa, particularly in complex multi-component protocols. When articles described multi-level combinations (e.g., “Si shenzhen” within “Naosanzhen”), the LLM sometimes failed to extract all hierarchical relationships. Original literature used diverse naming conventions (e.g., “Taiyang”, “EX-HN5”), and manual verification standardized these to GBT-12346-2021 codes. Quantitative efficacy data such as percentages and *p*-values were reliably extracted, but qualitative descriptions required human judgment for effect_level assignment. The manual verification step systematically corrected these errors before graph insertion, ensuring the final KG accurately represents the source literature while leveraging LLM efficiency with human expert quality control. We converted relevant Jin San Zhen literature and monograph content into continuous text via OCR and preprocessing, then used the Qwen3-MAX LLM with task-specific prompts to extract key information on diseases, main points, adjunct points, point locations, operational methods, treatment courses, efficacy, and experience summaries into standardized JSON structures. Each article produced an individual JSONL file, which, after manual review to remove invalid or duplicate data, was merged to form the final extraction dataset used for graph construction. We then used Neo4j as the graph database (27) to import the entities and relations and created uniqueness constraints and indexes on key attributes such as standardized acupoint codes and names, acupoint combination names, treatment plan IDs, and raw point names to avoid duplicate insertion and improve query efficiency based on names or codes.

### 2.1 KG intrinsic evaluation

**Figure 4:**
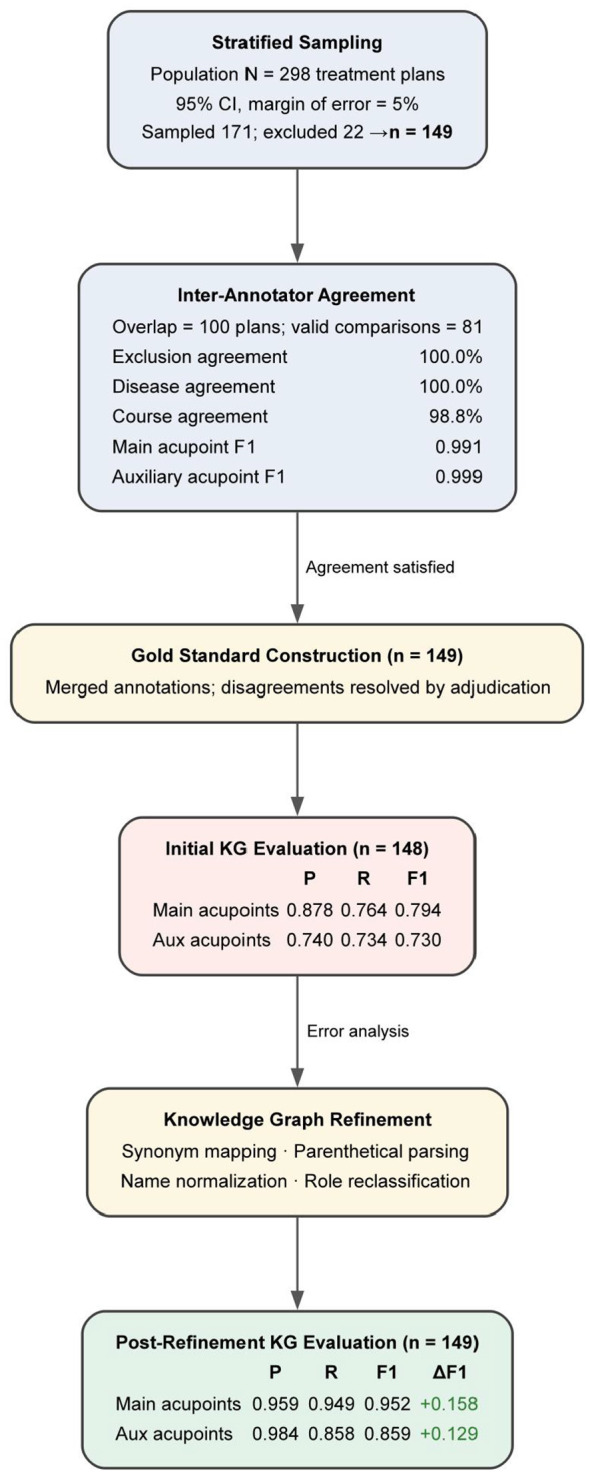
Knowledge graph annotation process.

To quantitatively evaluate the accuracy of the constructed KG, we designed a systematic intrinsic evaluation procedure using Precision (P), Recall (R), and F1 metrics against a human-annotated gold standard (Figure 4).

Gold standard construction. The KG contained *N* = 298 treatment plan records. We employed stratified random sampling to select a representative evaluation subset. Using a 95% confidence level and a 5% margin of error, the required sample size was calculated as 171. After initial sampling, 22 records were excluded because they originated exclusively from monograph content (where “main” vs. “auxiliary” acupoint distinctions are not applicable in the same manner as in clinical studies), yielding a final evaluation sample of *n* = 149 treatment plans.

Two annotators (C.J.J. and G.K.R.) independently annotated the sampled treatment plans by reading the original source articles and consulting the relevant monograph content. Because the KG was constructed from both clinical literature and authoritative monographs, the gold standard annotation likewise drew upon both sources, with the two mutually corroborating each other to strengthen annotation reliability. Specifically, for each plan, annotators recorded: (a) whether the record should be excluded, (b) the disease name, (c) the treatment course, (d) the set of main acupoints, and (e) the set of auxiliary acupoints. To assess inter-annotator reliability, 100 treatment plans were assigned to both annotators, of which 81 yielded valid paired comparisons (the remainder were excluded by both annotators or involved monograph-only content). Inter-annotator agreement was as follows: exclusion decisions, 100.0% agreement; disease names, 100.0% agreement; treatment course descriptions, 98.8% agreement; main acupoint sets, F1 = 0.991; auxiliary acupoint sets, F1 = 0.999. These high agreement values confirmed the reliability of the annotation process. Disagreements on the overlapping subset were resolved through discussion and adjudication to produce the final gold standard for the *n* = 149 evaluation plans.

Initial evaluation and error analysis. We compared the KG records against the gold standard for *n* = 148 plans (one plan was subsequently reclassified during adjudication). Error analysis revealed four major categories of discrepancies: (1) synonym mismatches, where different names referred to the same acupoint (e.g., “Taiyang” vs. “EX-HN5”); (2) parenthetical parsing failures, where acupoints listed within parentheses in the original text were missed during extraction; (3) name normalization inconsistencies, where variant spellings or abbreviations were not unified; and (4) role misclassification, where acupoints were incorrectly assigned as “main” instead of “auxiliary” or vice versa.

KG refinement. Based on the error analysis, we implemented four targeted refinement steps: (1) synonym mapping to merge equivalent acupoint names; (2) parenthetical parsing to capture acupoints listed in parenthetical expressions; (3) name normalization to standardize all acupoint names to GBT-12346-2021 codes; and (4) role reclassification to correct main/auxiliary assignments based on source text context.

Post-refinement evaluation. After refinement, the KG was re-evaluated against the full gold standard (*n* = 149). These results demonstrate that the refined KG achieves high fidelity in representing the acupoint information from source literature.

On this basis, we designed and implemented an intelligent QA system that integrates the KG with an LLM (28). Users can pose questions in natural language through a web interface, mobile client, or API, for example: “What are the core acupoint combinations in Jin San Zhen for treating stroke?” or “What clinical studies have reported the efficacy of Temporal Three Needles for pediatric cerebral palsy?”. On the backend, the system first invokes the LLM to parse the question, identify entities such as disease names, acupoint combination names, or specific acupoints, and classify the question into one of several predefined query types—for example, “retrieving plans by disease,” “retrieving diseases by plan,” “summarizing main/auxiliary points by disease,” or “retrieving related diseases by acupoint.” Next, based on the identified query type and entities, the system selects the corresponding Cipher template, fills the entities into the template, and generates Neo4j queries (29) to retrieve related diseases, treatment plans, acupoint combinations, specific acupoints, and their efficacy and usage frequencies as structured records. Because the disease and acupoint names in user queries may differ from the standardized names in the graph, the system introduces a string-similarity–based fuzzy matching mechanism during entity matching, with a predefined similarity threshold to filter candidate names, thereby improving tolerance to non-standard queries.

After graph retrieval is completed, the system feeds the original user question and the retrieved structured records into the LLM (30) to generate a fluent, well-organized, and academically styled Chinese answer. To minimize hallucinations and erroneous inferences, we explicitly instruct in the generation prompts that all conclusions, point combinations, and indications must be supported by the provided records, and that the model must not invent new diseases, prescriptions, or indications that do not exist in the KG. When the graph query returns no results, the system should inform the user that “no corresponding records are currently available in the graph” rather than attempting to fabricate an answer based on general medical knowledge. The generated answer is returned together with the structured records, enabling users to trace back to specific literature or monograph sources. The system also supports graphical visualization of local subgraphs to help users intuitively understand the point-selection logic of Jin San Zhen for specific conditions.

### 2.2 Tiered confidence generation strategy

Building on the template-based KG+LLM system described above, we developed an enhanced “hybrid” configuration that employs a tiered confidence generation strategy to address a fundamental limitation of strictly graph-constrained answer generation: when the KG does not contain information relevant to certain aspects of a user's question, the template-based system can only report “no corresponding records are currently available,” potentially leaving clinically relevant sub-questions unanswered. The tiered confidence generation strategy structures the LLM's answer into two explicitly labeled layers. The first layer (marked with a “High Confidence” indicator) is strictly and exclusively based on the structured records retrieved from the KG: the LLM is instructed that it must not add any acupoint names, combination names, disease names, or relationships that do not appear in the retrieved records. This layer preserves the same accuracy and traceability guarantees as the template-based approach. The second layer (marked with a “Supplementary, Not Verified by This KG” indicator) allows the LLM to draw upon its general TCM and acupuncture knowledge to address aspects of the question that the first layer could not cover. However, the second layer is subject to strict prohibitions: it must not fabricate Jin San Zhen–specific combination names or their acupoint compositions, must not invent specific acupoint prescriptions or needling parameters, must not cite non-existent literature, and must not claim that any information originates from the KG. Permitted supplementary content includes TCM syndrome differentiation reasoning and pathomechanism analysis, general treatment principles and combination rationale, general descriptions of clinical efficacy trends, introductions to commonly used assessment scales or outcome measures, and general safety and precautionary notes. This architectural design achieves a “1+1 >2” effect: the KG anchors the core clinical content with full traceability, while the LLM's general medical knowledge fills conceptual and contextual gaps that are beyond the scope of any finite knowledge base, with the explicit confidence labeling enabling users to appropriately weigh each component. The complete system prompt implementing this strategy is available in the source code repository.

### 2.3 QA system evaluation design

**Figure 5:**
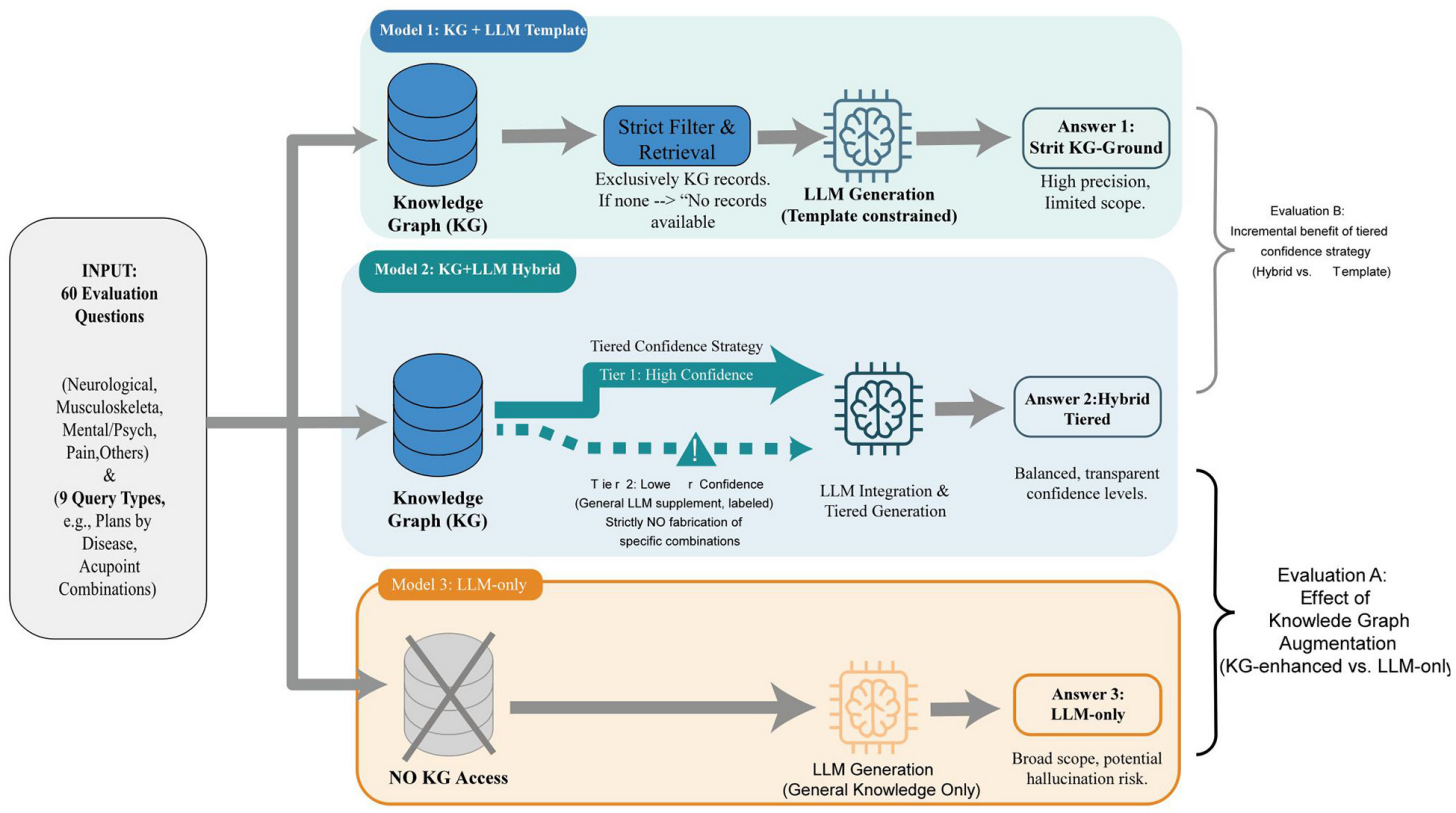
Schematic comparison of three models for medical question answering evaluation.

To evaluate the answer quality of the system (Figure 5), we constructed 60 evaluation questions covering different disease categories and the six predefined query types supported by the system (retrieving plans by disease, retrieving diseases by plan, summarizing main/auxiliary points by disease, retrieving related diseases by acupoint, retrieving acupoint combination details, and querying efficacy evidence). Questions were distributed across the major disease categories represented in the KG (neurological disorders, musculoskeletal conditions, mental/psychological disorders, pain conditions, and others) and were derived from typical clinical scenarios documented in the 191 included studies and the four authoritative monographs, representing the types of queries that primary care clinicians and trainees would most commonly pose in practice. For each question, we generated three answers from the system configurations described above: (1) the KG+LLM template model, (2) the KG+LLM hybrid model, and (3) the LLM-only model (31). Both KG-enhanced models share the same query pipeline—including LLM-based entity alignment, fuzzy matching, query type classification, Cipher template retrieval, and fallback search—and differ only in the answer generation prompt strategy. This three-way comparison allowed us to evaluate both the effect of KG augmentation (KG-enhanced vs. LLM-only) and the incremental benefit of the tiered confidence generation strategy (hybrid vs. template).

We invited two expert evaluators who were selected based on stringent criteria including at least 15 years of clinical acupuncture practice with proficiency in Jin San Zhen, direct training lineage from recognized Jin San Zhen masters, active involvement in Jin San Zhen clinical research and teaching, and advanced academic credentials in TCM acupuncture. Expert Y.J. (Yu Jin) serves as Director of the Jin San Zhen Research Center at Guangzhou University of Chinese Medicine with 18 years of clinical experience and has trained directly under Professor Jin Rui, while Expert C.J. (Chen Jianchao) holds a Doctorate in Acupuncture and Chinese Medicine with 15 years of international clinical practice in both Australia and China and completed advanced Jin San Zhen training programs. While larger expert panels are ideal, the pool of individuals meeting all qualification criteria is limited in China, and a two-expert design is methodologically acceptable when highly qualified domain experts are scarce provided their ratings demonstrate good agreement.

We assessed inter-rater agreement quantitatively: across all 60 questions × 3 dimensions × 3 systems = 540 paired ratings, the two experts showed exact agreement on 83.89% of ratings and agreement within ±1 point on 99.44% of ratings; the mean absolute difference between raters was 0.0315 points on the 5-point scale. These descriptive statistics demonstrate high scoring consistency. The evaluation combined subjective expert ratings with objective verification: for each question, we pre-compiled correct answers based on authoritative monographs to verify factual accuracy, specifically checking for false positive claims (acupoint combinations, disease indications, or efficacy claims not supported by any documented source) and unverifiable statements. Additionally, we adopted a double-blind design in which the experts were not informed which system produced which answer, and the three answers for each question were presented in randomized order.

## 3 Results

### 3.1 KG scale and structure

**Figure 6:**
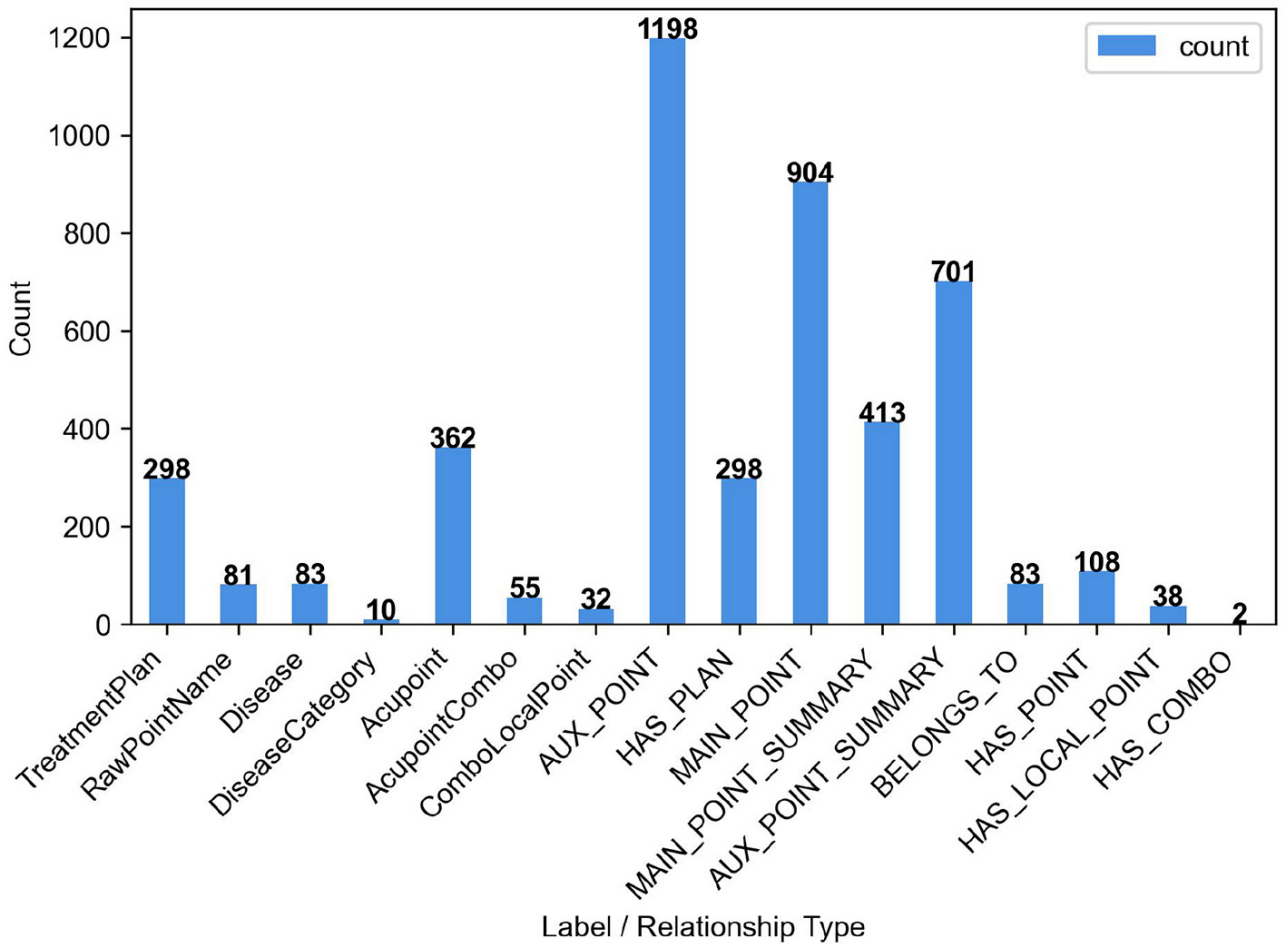
Entity and relationship quantity statistics chart.

Statistical results showed that the final KG comprised 921 nodes and 3,745 relations, including 362 Acupoint nodes, 298 TreatmentPlan nodes, 81 RawPointName nodes, 83 Disease nodes, 55 AcupointCombo nodes (including the 46 core three-needle prescriptions from the monographs and 9 additional combinations identified in the clinical literature), and 32 ComboLocalPoint nodes. At the relation level, there were 904 MAIN_POINT relations, 413 MAIN_POINT_SUMMARY relations, 1,198 AUX_POINT relations, 701 AUX_POINT_SUMMARY relations, 298 HAS_PLAN relations, 108 HAS_POINT relations, and several HAS_LOCAL_POINT and HAS_COMBO relations (Figure 6). Together, these systematically represent the prescription structures and acupoint usage patterns of Jin San Zhen therapy across different diseases.

### 3.2 KG intrinsic evaluation results

The intrinsic evaluation demonstrated both high inter-annotator reliability and high KG accuracy. Inter-annotator agreement on the 81 valid overlapping annotations was excellent: exclusion decisions showed 100.0% agreement, disease names showed 100.0% agreement, treatment course descriptions showed 98.8% agreement, and the F1 scores for main and auxiliary acupoint sets were 0.991 and 0.999, respectively.

The initial KG evaluation (*n* = 148) revealed moderate accuracy before refinement, with F1 = 0.794 for main acupoints (*P* = 0.878, *R* = 0.764) and F1 = 0.730 for auxiliary acupoints (*P* = 0.740, *R* = 0.734). Error analysis identified four systematic error categories: synonym mismatches, parenthetical parsing failures, name normalization inconsistencies, and main/auxiliary role misclassification. After targeted refinement addressing these errors, the post-refinement evaluation (*n* = 149) showed substantial improvement: main acupoint F1 increased to 0.952 (*P* = 0.959, *R* = 0.949; Δ F1 = +0.158), and auxiliary acupoint F1 increased to 0.859 (*P* = 0.984, *R* = 0.858; Δ F1 = +0.129). These results confirm that the KG achieves high fidelity in representing the acupoint selection information documented in the source literature.

### 3.3 QA system expert evaluation results

For each of the 60 questions, both experts independently scored the three system answers on correctness, professionalism, and completeness using a 1–5 scale. Statistical analysis using paired *t*-tests revealed a clear and consistent performance hierarchy across all dimensions (Table 1).

**Table 1:** Intergrity, professionalism, and correctness of *t*-test analysis.

Metric	Model 1	Model 2	Mean 1	Mean 2	Mean_Diff	T_Statistic	*P*_Comparison
Correctness	QA + LLM	OnlyLLM	4.75	4.05	0.7	15.75206842	*p* < 0.01
Correctness	QA + LLM	QA + LLM (hybrid)	4.75	5	−0.25	−6.298147876	*p* < 0.01
Correctness	OnlyLLM	QA + LLM (hybrid)	4.05	5	−0.95	−47.54997371	*p* < 0.01
Professional	QA + LLM	OnlyLLM	4.766666667	3.65	1.116666667	19.10847904	*p* < 0.01
Professional	QA + LLM	QA + LLM (hybrid)	4.766666667	5	−0.233333333	−6.018088675	*p* < 0.01
Professional	OnlyLLM	QA + LLM (hybrid)	3.65	5	−1.35	−30.87568124	*p* < 0.01
Integrity	QA + LLM	OnlyLLM	4.025	4.116666667	−0.091666667	−2.80122589	*p* < 0.01
Integrity	QA + LLM	QA + LLM (hybrid)	4.025	4.4	−0.375	−7.955995999	*p* < 0.01
Integrity	OnlyLLM	QA+LLM (hybrid)	4.116666667	4.4	−0.283333333	−5.277022225	*p* < 0.01

For correctness, the KG+LLM hybrid model achieved a mean score of 5.00, significantly higher than the KG+LLM template model (4.75, *t* = −6.30, *p* < 0.01) and the LLM-only model (4.05, *t* = −47.55, *p* < 0.01). The template model also significantly outperformed the LLM-only model (*t*= 15.75, *p* < 0.01). For professionalism, the hybrid model again achieved a mean score of 5.00, compared with 4.77 for the template model (*t* = −6.02, *p* < 0.01) and 3.65 for the LLM-only model (*t* = −30.88, *p* < 0.01); the template model significantly outperformed the LLM-only model (*t* = 19.11, *p* < 0.01). For completeness, the hybrid model scored 4.40, significantly higher than both the template model (4.03, *t* = −7.96, *p* < 0.01) and the LLM-only model (4.12, *t* = −5.28, *p* < 0.01). The template model scored slightly lower than the LLM-only model on completeness (4.03 vs. 4.12, *t* = −2.80, *p* < 0.01), though the mean difference of 0.09 points represents a negligible effect size.

These results demonstrate three key findings. First, augmenting the LLM with KG retrieval substantially improves correctness (Δ = +0.70) and professionalism (Δ = +1.12), confirming the value of structured evidence grounding. Second, the tiered confidence generation strategy in the hybrid model further elevates performance across all three dimensions, achieving perfect or near-perfect scores. Third, the hybrid model resolves the completeness trade-off inherent in strictly graph-constrained generation: the template model scored slightly below the LLM-only model on completeness (4.03 vs. 4.12), whereas the hybrid model achieved the highest completeness (4.40) by supplementing graph-grounded content with labeled general TCM knowledge while the first-tier content ensures accuracy and traceability, demonstrating a synergistic effect across all three dimensions.

According to experts' subjective feedback, the hybrid model's layered presentation was particularly valued: the clear visual distinction between graph-verified content and supplementary general knowledge allowed readers to immediately identify which statements carry evidence-based support and which require further verification against authoritative sources. Expert Y.J. commented that “The tiered format is ideal for clinical reference—I can trust the graph-derived content directly and treat the supplementary layer as helpful context that I would verify before acting upon.” In contrast, answers from the LLM-only model, while sometimes appearing more elaborate, lacked any such confidence differentiation, making it impossible to distinguish evidence-supported claims from potentially fabricated content without independent verification.

### 3.4 Hallucination and false positive analysis

A key design goal of integrating the KG with the LLM was to reduce factual errors and unsupported claims commonly termed “hallucinations” in LLM-generated content. We evaluated this across all 60 evaluation questions by verifying the factual correctness of generated answers against authoritative Jin San Zhen monographs that served as gold-standard sources.

Both KG-enhanced systems achieved zero false positive claims. For the KG ± LLM template model (0/60, 100% factual accuracy), this result is expected given that answer generation is strictly limited to graph-retrieved records. For the KG ± LLM hybrid model (0/60, 100% factual accuracy), this result validates the effectiveness of the tiered confidence generation strategy's architectural safeguards: while the second tier permits supplementary general knowledge, the strict prohibitions against fabricating Jin San Zhen–specific combination names, acupoint compositions, needling parameters, and literature citations successfully prevented hallucinations in the supplementary layer. The supplementary content generated by the hybrid model consisted exclusively of permitted categories—TCM syndrome differentiation reasoning, general treatment principles, assessment scale introductions, and safety notes—none of which introduced factually incorrect Jin San Zhen–specific claims. In contrast, the LLM-only model contained multiple instances of unverifiable or incorrect claims across the 60 questions, including invented combination names not documented in any source and misattributed acupoint properties, while providing no citations for independent verification. All answers from both KG-enhanced models included specific bibliographic references enabling independent verification, with each graph-derived claim traceable to particular records in the source literature. The hybrid model additionally provided explicit confidence labeling, allowing users to distinguish which portions of the answer are directly supported by the KG (high confidence) and which represent *supplement*ary general knowledge requiring further verification (lower confidence). This transparency mechanism represents a practical safeguard against over-reliance on any single information source.

Regarding false negatives, no formal analysis was conducted at the QA level, as both KG-enhanced systems retrieve all matching records from the KG for a given query. Nevertheless, the expert evaluation results provide indirect evidence regarding QA-level retrieval completeness. Across all 60 evaluation questions that targeted diseases and acupoint combinations present in the KG, the system successfully retrieved relevant graph records in every case, and both experts' high correctness scores (hybrid model: 5.00; template model: 4.75) indicate that no clinically significant retrieval failures occurred at the query level. However, because the evaluation design primarily assessed answer quality rather than conducting exhaustive record-level recall audits—that is, experts rated overall answer correctness rather than verifying whether every individual matching record in the KG was returned for each query—we cannot definitively exclude minor record-level omissions that did not affect overall answer quality. Additionally, we tested queries involving diseases not represented in the KG; in all such cases, the system correctly reported the absence of relevant records rather than fabricating responses, demonstrating appropriate out-of-scope query handling (i.e., true negatives) rather than constituting a false negative analysis *per se*. The KG intrinsic evaluation provides relevant data: the post-refinement Recall of 0.949 for main acupoints indicates that approximately 5% of main acupoints documented in source literature may be missed in the graph itself. The hybrid model's second-tier supplementary knowledge partially compensates for such gaps at the answer level by providing general contextual information, though it cannot substitute for specific graph records that are absent. These results, while encouraging, should be interpreted cautiously given the sample size of 60 questions; larger-scale validation is warranted, as a larger question set could reveal edge cases where the second-tier prohibitions are insufficient.

Regarding patient safety implications, the consistent zero false positive rate across both KG-enhanced configurations means no incorrect acupoint combinations or contraindicated treatment plans were generated, whereas the false positives in the LLM-only system could potentially mislead clinical decisions if acted upon without independent verification.

## 4 Overall contribution

Overall, this study represents the first systematic integration of nearly a decade of Chinese clinical research evidence and the core content of authoritative Jin San Zhen monographs to construct a specialized KG, and, on this basis, to develop and iteratively optimize an intelligent QA system for TCM acupuncture clinical and educational settings (32). The comparison of three system configurations demonstrates that KG augmentation substantially improves correctness and professionalism over general-purpose LLMs, and that the tiered confidence generation strategy—which combines strict graph-grounded answers with explicitly labeled supplementary general knowledge—further resolves the completeness limitation of strictly graph-constrained generation while maintaining zero false positive rates. This complementary synergy, in which the KG provides an authoritative evidence anchor and the LLM fills conceptual and contextual gaps with appropriate confidence labeling, offers a practical architectural pattern for medical AI systems that must balance accuracy, completeness, and transparency. The RAG-based technical route, together with the tiered confidence generation approach demonstrated here, provides a replicable paradigm for the digital curation and intelligent application of other characteristic TCM therapies, such as acupoint embedding, auricular acupuncture, and fire needling (33). For primary care and general practitioners, this system has the potential to serve as an auxiliary tool for rapidly accessing information on Jin San Zhen indications, core three-needle combinations, routine treatment courses, and existing evidence-based conclusions (34), with the explicit confidence labeling supporting more informed evaluation of the information provided (35).

## 5 Discussion

This study nonetheless has several limitations that warrant careful interpretation of our findings. Our evaluation employed 60 test questions and two expert raters across three system configurations, providing 540 paired ratings that enabled detection of statistically significant differences across all pairwise comparisons and all three evaluation dimensions. However, this sample may not fully capture the diversity of real-world clinical queries (36). The KG+LLM hybrid model achieved ceiling effects on correctness and professionalism (mean = 5.00 from both raters across all 60 questions), which, while indicating excellent performance, limits the ability to detect further nuanced quality differences and may partly reflect the 5-point scale's resolution rather than truly flawless answers. Future validation should employ larger question sets (≥100 questions), additional expert raters including primary care physicians (the intended end users), finer-grained scoring scales (e.g., 1–10) to mitigate ceiling effects, and multi-center evaluation to improve generalizability. Regarding the representativeness of the 60 evaluation questions, while they were systematically constructed to cover the full range of query types and disease categories in the KG, they were not derived from a formal survey of end users. Future studies should collect authentic queries from real clinical users to better represent the diversity and complexity of actual information needs.

A notable finding of this study is that the tiered confidence generation strategy effectively resolved the completeness limitation inherent in strictly graph-constrained answer generation. The template-based KG+LLM model scored lower on completeness than even the LLM-only model, reflecting a design trade-off where rigid adherence to graph-retrieved records ensures accuracy and traceability but necessarily omits contextual information that falls outside the scope of the structured KG. The tiered approach addresses this by introducing a clearly labeled second layer that draws on the LLMs general TCM knowledge for precisely these conceptual and contextual gaps, while maintaining strict prohibitions against fabricating domain-specific clinical claims (e.g., Jin San Zhen combination names, acupoint compositions, needling parameters, and literature citations). This synergistic benefit is reflected in the hybrid model's simultaneously highest scores across all three evaluation dimensions. As Expert Y.J. noted during post-evaluation feedback, “The hybrid model combines the rigor I need for clinical reference with the contextual richness that aids understanding—the tiered labeling lets me immediately know which parts I can cite directly and which parts I should treat as general background” (37).

Both expert raters are actively involved in Jin San Zhen research and clinical practice, and while this ensures qualified evaluation, it may introduce preference for structured, evidence-anchored discourse characteristic of the KG-enhanced systems. The ceiling effects observed in hybrid model ratings (correctness and professionalism both 5.00 across all 60 questions from both raters) warrant particular scrutiny. While this may reflect genuine excellence of the tiered generation approach, it could also indicate that experts familiar with the source literature recognized specific citations in the graph-derived layer and preferentially rewarded answers that explicitly referenced known studies. Additionally, the clear visual formatting of the tiered answer may have introduced a presentation bias despite the double-blind design, as this distinctive format could potentially be associated with a specific system. Future evaluation should incorporate external expert raters unfamiliar with the project, primary care physicians representing the target user population, answer presentation normalization to eliminate formatting cues, objective accuracy metrics beyond subjective ratings, and prospective field trials measuring impact on clinical decision quality and patient outcomes.

The current QA system employs predefined query type classification (six types) and template-based Cipher generation, which effectively constrains LLM generation to graph-supported content and substantially reduces hallucination but inherently limits flexibility when processing highly ambiguous, multi-intent, or open-ended reasoning queries. During development testing, we observed that the system handles straightforward clinical queries well (e.g., “What are some commonly used Jin San Zhen (a traditional Chinese medicine) treatment methods for stroke sequelae?”), but struggles with complex compound queries that involve multiple clinical considerations simultaneously (e.g., “How should elderly patients with post-stroke sequelae and hypertension be treated with Jin's Three-Needle acupuncture and combined with medication?”). In such cases, the system currently addresses the primary query component but may not fully address all aspects of the question. Future enhancements will focus on query decomposition to break complex questions into atomic sub-queries that can be addressed sequentially, multi-turn dialogue capability to actively request clarification from users when queries are ambiguous, and hybrid retrieval combining structured graph queries with semantic search over unstructured text fields stored in the knowledge base. All enhancements will be validated to ensure expanded functionality does not compromise factual accuracy and evidence traceability, which remain the system's core design principles.

Future work may proceed in several directions. First, we will continue to expand data sources by incorporating studies from additional databases such as CBM, PubMed, and Web of Science, thereby covering broader populations, disease spectra, and study designs to enhance the graph's representativeness and international applicability. Second, we plan to explore integration with real-world clinical data (38), for example by interfacing with electronic health record systems or regional health data platforms, and under appropriate privacy and ethical safeguards, to evaluate the system's influence on physicians' decision-making behavior and patient outcomes in routine clinical workflows. Third, building on the current KG, we aim to gradually incorporate individual patient characteristics—such as age, constitution, comorbidities, and previous treatment responses—to explore the development of a Jin San Zhen prescription recommendation system with a degree of individualization, thereby providing methodological support for future personalized and precision acupuncture.

We envision three implementation modes: a standalone web/mobile consultation tool for point-of-care queries (39) (typical response time < 5 s), a passive EMR-integrated suggestion module, and an interactive educational tool for clinical training and continuing education. The system is designed with explicit clinical autonomy principles: every recommendation includes bibliographic references for independent verification, all content remains fully editable by the clinician, and conflicting or limited evidence is explicitly disclosed rather than concealed. Importantly, the system is intended exclusively for healthcare professional use and displays prominent disclaimers accordingly; any future patient-facing adaptation would require substantial content redesign, additional safety guardrails, and regulatory compliance review. A feedback mechanism enables users to report inaccuracies for continuous quality improvement. Detailed implementation specifications are available in the source code repository.

## Data Availability

The datasets presented in this study can be found in online repositories. The names of the repository/repositories and accession number(s) can be found below: https://github.com/ChenJunjie0906/SanzhenQ-A/tree/main.
