# Supplementary material for: Construction and evaluation of the knowledge graph and large model question-answering system for Jin San Zhen therapy: a tool study for primary care and general practice
Source: Front Med (Lausanne). 2026 Apr 9;13:1755583. doi: 10.3389/fmed.2026.1755583 (PMC13103959; doi:10.3389/fmed.2026.1755583)
Supplement: Supplementary file 1 [file Supplementary_file_1.docx]

## 1. System Architecture Overview

The system follows a Retrieval-Augmented Generation (RAG) architecture comprising three layers.

**API Layer.** A FastAPI-based RESTful backend exposes the primary endpoint POST /api/qa, which accepts a natural language question, optional conversation history, and configuration options (e.g., whether to enable knowledge graph retrieval, maximum number of treatment plans to return). Responses are returned as structured JSON objects (QAResponse) containing the generated natural language answer, the classified query type, extracted entities, the executed Cypher query, raw retrieved records, entity alignment suggestions, and an answer_source label ("kg_hybrid" or "llm_only"). A health check endpoint (GET /health) is also provided. Cross-Origin Resource Sharing (CORS) middleware is enabled for frontend integration.

**Knowledge Graph Layer.** Neo4j serves as the graph database, storing six entity types (Disease, TreatmentPlan, Acupoint, AcupointCombo, ComboLocalPoint, RawPointName) and eight relationship types (HAS_PLAN, MAIN_POINT, AUX_POINT, HAS_POINT, HAS_LOCAL_POINT, HAS_COMBO, MAIN_POINT_SUMMARY, AUX_POINT_SUMMARY), totaling 921 nodes and 3,745 relationships. The graph supports CALL {} subquery syntax used in several query templates.

**LLM Service Layer.** The system invokes Alibaba Cloud DashScope's model via an OpenAI-compatible SDK interface. Two independent model configuration parameters (QWEN_MODEL_PARSE for question parsing and entity alignment, QWEN_MODEL_ANSWER for answer generation) allow different model versions to be assigned to different tasks. All LLM interactions are performed through a unified helper function (_chat_completion) that wraps the API call with model name, message list, and temperature parameters.

## 2. Data Model

The request schema (QARequest) includes four fields: question (required, the user's natural language query), history (optional, a list of prior user/assistant message pairs for multi-turn dialogue), options (optional, containing max_plans with a default of 10 and language defaulting to Chinese), and use_kg (optional, boolean, defaulting to True).

The response schema (QAResponse) includes seven fields: answer (the generated natural language answer), query_type (the classified query type string), entities (a dictionary mapping entity categories to name lists), cypher (the executed Cypher query string), records (raw retrieved records as a list of dictionaries), entity_suggest (alignment suggestions for unmatched entities), and answer_source (either "kg_hybrid" or "llm_only").

## 3. Complete Processing Pipeline

A user question traverses the following steps from input to output.

### Step 1: Question Parsing (call_llm_for_parse)

The user's question, together with any conversation history, is sent to the LLM with a system prompt that instructs it to output a strict JSON structure containing four fields: query_type (one of ten predefined types), diseases (list of disease names), combos (list of Jin San Zhen combination names), and points (list of individual acupoint names).

The system prompt defines ten query types: disease_to_plans (retrieve treatment plans for a disease), combo_to_diseases (retrieve diseases treatable by a combination), disease_combo_to_effect (retrieve efficacy evidence for a specific disease-combination pair), point_to_combos (retrieve combinations and diseases associated with an acupoint), disease_to_point_summary (retrieve high-frequency main/auxiliary acupoint summaries for a disease), combo_to_points (retrieve acupoint composition of a combination), disease_compare_plans (compare treatment plans for the same disease), combo_detail (retrieve comprehensive details of one or more combinations), multi_disease_common (identify shared combinations/acupoints across multiple diseases), and unknown (unclassifiable queries).

The prompt includes explicit multi-turn coreference resolution instructions: when the user employs pronouns (e.g., "it," "this combination") or elides the subject, the LLM must infer the referent from conversation history and populate the corresponding entity fields accordingly.

If the LLM call fails, the system falls back to keyword-based heuristic rules (e.g., detecting "颞三针" in the query text and mapping to combo_to_diseases).

### Step 2: Three-Tier Entity Alignment (normalize_entities)

Because user-provided entity names frequently differ from standardized names in the knowledge graph, the system employs a three-tier progressive alignment strategy.

**Tier 1 — Exact Match.** User entities are compared against the full set of standardized names cached from Neo4j (see Section 5 for caching Cypher queries). The cache uses a time-limited LRU strategy with a 600-second TTL to balance freshness and performance.

**Tier 2 — LLM Semantic Alignment (**_llm_entity_align**).** Entities not matched in Tier 1 are sent to the LLM along with the complete candidate list for each entity category. The LLM is instructed to identify semantic equivalences based on TCM and Western medicine terminology (e.g., "中风" → "脑卒中后遗症," "面瘫" → "周围性面瘫") and must only return names that exist in the candidate list. The result is a JSON mapping from user names to standardized names (or null if no match exists).

**Tier 3 — String Similarity Fallback (**_best_match**).** Entities still unmatched after Tier 2 are processed using Python's difflib.get_close_matches with a similarity cutoff of 0.5 and a maximum of 5 candidates. The best match is accepted only if its SequenceMatcher ratio meets the threshold.

Entities that fail all three tiers are retained as-is, and the system populates the entity_suggest field with unmatched entity information and candidate lists, enabling the frontend to present suggestions to the user.

### Step 3: Query Type Auto-Adjustment (_adjust_query_type)

When the parsed query_type is incompatible with the actually extracted entities (e.g., disease_to_plans but no diseases were extracted while combos are present), the system automatically downgrades the query type to match the available entities. The adjustment rules follow a priority logic: if the primary entity category for a query type is empty, the system attempts to reclassify based on which entity categories are populated, defaulting to unknown when no entities are available. All adjustments are logged for debugging.

### Step 4: Cypher Template Construction and Execution (build_and_run_query)

For each query type, the system selects and parameterizes a pre-defined Cypher template. All templates use parameterized queries ($disease_name, $combo_name, $point_name, $limit) to prevent injection and ensure reusability. The complete set of Cypher templates is presented in Section 6.

### Step 5: Empty-Result Fallback Search (_fallback_search)

If the primary query returns no results (typically due to residual entity misalignment), the system initiates a fuzzy fallback search using CONTAINS substring matching. For disease-related query types, the system progressively shortens the keyword (full name → first three characters → first two characters) to broaden recall. Fallback results are explicitly flagged in the final answer with a warning prefix. The complete set of fallback Cypher templates is presented in Section 7.

### Step 6: Record Cleaning (_clean_records)

Raw records from Neo4j are cleaned before being passed to the LLM: fields with None values or empty strings are removed, lists are filtered to exclude None elements and empty dictionaries, and rows with no remaining valid fields are discarded entirely. This ensures the JSON payload sent to the LLM is compact and unambiguous.

### Step 7: Tiered Confidence Answer Generation (call_llm_for_answer)

This step implements the "tiered confidence generation strategy" described in the manuscript. The LLM receives a detailed system prompt requiring that the answer be structured into two explicitly labeled layers.

The first layer (marked with a "High Confidence" indicator) must be generated strictly and exclusively from the records JSON provided. The LLM is prohibited from adding any acupoint names, combination names, disease names, or relationships not present in the records. The system prompt specifies layer-one emphasis points for each query type: disease_to_plans should summarize main acupoint combinations, auxiliary acupoints, electroacupuncture/moxibustion/drug adjuncts, treatment courses, and efficacy conclusions; combo_to_diseases should list diseases treated; disease_combo_to_effect should describe study design, course, and key efficacy indicators; point_to_combos should summarize which combinations include the acupoint and which diseases they treat; disease_to_point_summary should list high-frequency main and auxiliary acupoints; combo_to_points should detail standard acupoints, local points, and sub-combinations; disease_compare_plans should compare efficacy across plans; combo_detail should present complete composition, needling methods, indications, and contrastive analysis when multiple combinations are queried; and multi_disease_common should identify shared combinations and acupoints.

The second layer (marked with a "Supplementary, Not Verified by This Knowledge Graph" indicator) permits the LLM to draw on its general TCM and acupuncture knowledge for aspects the first layer could not cover, subject to four strict prohibitions: no fabricating Jin San Zhen–specific combination names or their acupoint compositions, no inventing specific acupoint prescriptions or needling parameters, no citing non-existent literature, and no claiming information originates from the knowledge graph. Permitted content categories are explicitly enumerated: TCM syndrome differentiation reasoning and pathomechanism analysis, general treatment principles and combination rationale, general descriptions of clinical efficacy trends, introductions to commonly used assessment scales, and general safety and precautionary notes. If the first layer fully answers all aspects of the question, the second layer is not generated.

When records is empty, the system passes an explicit text placeholder—"No matching records were retrieved from the knowledge graph for this query; the first layer should state this situation, and the second layer should provide supplementary information"—rather than hard-coding a refusal, allowing the prompt to naturally guide the layered response.

All answers conclude with a mandatory disclaimer.

## 4. Three System Configurations Compared in the Evaluation

The three system configurations evaluated in the manuscript differ as follows.

The KG+LLM template model is a configuration where the generation prompt strictly constrains the LLM to answer only based on retrieved records, with a hard-coded early return of "no records currently available" when records are empty. No second-layer supplementation is permitted.

The KG+LLM hybrid model corresponds to the current system implementing the tiered confidence generation strategy described above. It shares the identical query pipeline (Steps 1–6) with the template model and differs only in the Step 7 generation prompt. The answer_source field is set to "kg_hybrid".

The LLM-only model corresponds to the use_kg=False branch, which invokes call_llm_for_answer_no_kg and bypasses knowledge graph retrieval entirely. The LLM answers based solely on its pre-trained knowledge, with a system prompt indicating that no graph data is available. The answer_source field is set to "llm_only".

This design ensures fair comparison: both KG-enhanced models use the same retrieval pipeline, differing only in generation strategy; the LLM-only model operates independently.

## 5. Entity Caching Cypher Queries

Three queries are used to populate the in-memory entity caches, each executed at most once per 600-second TTL interval.

All diseases:

cypher

MATCH (d:Disease)RETURN DISTINCT d.name AS name

**All acupoint combinations:**

cypher

MATCH (c:AcupointCombo)RETURN DISTINCT c.name AS name

**All acupoints:**

cypher

MATCH (a:Acupoint)RETURN DISTINCT a.name AS name

## 6. Primary Query Cypher Templates

### 6.1 disease_to_plans

This query type uses two sequential queries. The first retrieves all treatment plans for a disease along with their complete acupoint structure via ten CALL {} subqueries:

Cypher

```

MATCH (d:Disease {name: $disease_name})-[:HAS_PLAN]->(p:TreatmentPlan)WITH d, p ORDER BY p.plan_id LIMIT $limit

CALL {

WITH p

OPTIONAL MATCH (p)-[:MAIN_POINT]->(mc:AcupointCombo)

RETURN collect(DISTINCT mc.name) AS main_combos

}

CALL {

WITH p

OPTIONAL MATCH (p)-[:AUX_POINT]->(ac:AcupointCombo)

RETURN collect(DISTINCT ac.name) AS aux_combos

}

CALL {

WITH p

OPTIONAL MATCH (p)-[:MAIN_POINT]->(mp:Acupoint)

RETURN collect(DISTINCT mp.name) AS main_points

}

CALL {

WITH p

OPTIONAL MATCH (p)-[:AUX_POINT]->(ap:Acupoint)

RETURN collect(DISTINCT ap.name) AS aux_points

}

CALL {

WITH p

OPTIONAL MATCH (p)-[:MAIN_POINT]->(mc2:AcupointCombo)-[:HAS_POINT]->(msp:Acupoint)

RETURN collect(DISTINCT msp.name) AS main_combo_std_points

}

CALL {

WITH p

OPTIONAL MATCH (p)-[:MAIN_POINT]->(mc3:AcupointCombo)-[:HAS_LOCAL_POINT]->(mlp:ComboLocalPoint)

RETURN collect(DISTINCT mlp.name) AS main_combo_local_points

}

CALL {

WITH p

OPTIONAL MATCH (p)-[:MAIN_POINT]->(mc4:AcupointCombo)-[:HAS_COMBO]->(msc:AcupointCombo)

RETURN collect(DISTINCT msc.name) AS main_combo_sub_combos

}

CALL {

WITH p

OPTIONAL MATCH (p)-[:AUX_POINT]->(ac2:AcupointCombo)-[:HAS_POINT]->(asp:Acupoint)

RETURN collect(DISTINCT asp.name) AS aux_combo_std_points

}

CALL {

WITH p

OPTIONAL MATCH (p)-[:AUX_POINT]->(ac3:AcupointCombo)-[:HAS_LOCAL_POINT]->(alp:ComboLocalPoint)

RETURN collect(DISTINCT alp.name) AS aux_combo_local_points

}

CALL {

WITH p

OPTIONAL MATCH (p)-[:AUX_POINT]->(ac4:AcupointCombo)-[:HAS_COMBO]->(asc:AcupointCombo)

RETURN collect(DISTINCT asc.name) AS aux_combo_sub_combos

}

```

RETURN

d.name AS disease,

p.plan_id AS plan_id,

p.method_text AS method_text,

p.course_text AS course_text,

p.effect_text AS effect_text,

p.position_text AS position_text,

p.effect_level AS effect_level,

p.has_electroacupuncture AS has_electroacupuncture,

p.has_moxibustion AS has_moxibustion,

p.has_drug AS has_drug,

main_combos, aux_combos, main_points, aux_points,

main_combo_std_points, main_combo_local_points, main_combo_sub_combos,

aux_combo_std_points, aux_combo_local_points, aux_combo_sub_combos

The second query retrieves the disease-level high-frequency acupoint summaries:

Cypher

```

MATCH (d:Disease {name: $disease_name})OPTIONAL MATCH (d)-[:MAIN_POINT_SUMMARY]->(mp)

OPTIONAL MATCH (d)-[:AUX_POINT_SUMMARY]->(ap)

RETURN

collect(DISTINCT CASE WHEN mp:Acupoint THEN mp.name END) AS disease_main_points,

collect(DISTINCT CASE WHEN mp:AcupointCombo THEN mp.name END) AS disease_main_combos,

collect(DISTINCT CASE WHEN ap:Acupoint THEN ap.name END) AS disease_aux_points,

collect(DISTINCT CASE WHEN ap:AcupointCombo THEN ap.name END) AS disease_aux_combos

```

The summary fields are merged into each plan record before being passed to the LLM, providing both plan-level detail and disease-level statistical context.

### 6.2 combo_to_diseases

Cypher

```

MATCH (c:AcupointCombo {name: $combo_name})OPTIONAL MATCH (d:Disease)-[:HAS_PLAN]->(p:TreatmentPlan)

WHERE (p)-[:MAIN_POINT]->(c) OR (p)-[:AUX_POINT]->(c)

RETURN

c.name AS combo_name,

c.indications AS combo_indications,

c.acupuncture_method_json AS combo_acu_method,

collect(DISTINCT d.name) AS diseases,

collect(DISTINCT p.plan_id) AS plan_ids

```

### 6.3 disease_combo_to_effect

Cypher

```

MATCH (d:Disease {name: $disease_name})-[:HAS_PLAN]->(p:TreatmentPlan)MATCH (c:AcupointCombo {name: $combo_name})

WHERE (p)-[:MAIN_POINT]->(c) OR (p)-[:AUX_POINT]->(c)

RETURN

d.name AS disease, c.name AS combo_name,

p.plan_id AS plan_id, p.method_text AS method_text,

p.course_text AS course_text, p.effect_text AS effect_text,

p.position_text AS position_text, p.effect_level AS effect_level,

p.has_electroacupuncture AS has_electroacupuncture,

p.has_moxibustion AS has_moxibustion, p.has_drug AS has_drug

LIMIT $limit

```

### 6.4 point_to_combos

Cypher

```

MATCH (a:Acupoint {name: $point_name})OPTIONAL MATCH (combo:AcupointCombo)-[:HAS_POINT]->(a)

OPTIONAL MATCH (p_main:TreatmentPlan)-[:MAIN_POINT]->(a)

OPTIONAL MATCH (d_main:Disease)-[:HAS_PLAN]->(p_main)

OPTIONAL MATCH (p_aux:TreatmentPlan)-[:AUX_POINT]->(a)

OPTIONAL MATCH (d_aux:Disease)-[:HAS_PLAN]->(p_aux)

RETURN

a.name AS point_name,

collect(DISTINCT combo.name) AS combos,

collect(DISTINCT d_main.name) AS diseases_as_main,

collect(DISTINCT d_aux.name) AS diseases_as_aux,

count(DISTINCT p_main) AS main_plan_count,

count(DISTINCT p_aux) AS aux_plan_count

```

### 6.5 disease_to_point_summary

cypher

```

MATCH(d:Disease{name:$disease_name})OPTIONALMATCH (d)-[:MAIN_POINT_SUMMARY]->(mp)

OPTIONAL MATCH (d)-[:AUX_POINT_SUMMARY]->(ap)

RETURN

d.name AS disease,

collect(DISTINCT CASE WHEN mp:Acupoint THEN mp.name END) AS main_points,

collect(DISTINCT CASE WHEN mp:AcupointCombo THEN mp.name END) AS main_combos,

collect(DISTINCT CASE WHEN ap:Acupoint THEN ap.name END) AS aux_points,

collect(DISTINCT CASE WHEN ap:AcupointCombo THEN ap.name END) AS aux_combos

```

### 6.6 combo_to_points

Cypher

```

MATCH (c:AcupointCombo {name: $combo_name})CALL {

WITH c

OPTIONAL MATCH (c)-[r1:HAS_POINT]->(stdPoint:Acupoint)

RETURN collect(DISTINCT {name: stdPoint.name, needle_method: r1.needle_method}) AS std_points

}

CALL {

WITH c

OPTIONAL MATCH (c)-[r2:HAS_LOCAL_POINT]->(localPoint:ComboLocalPoint)

RETURN collect(DISTINCT {name: localPoint.name, needle_method: r2.needle_method}) AS local_points

}

CALL {

WITH c

OPTIONAL MATCH (c)-[:HAS_COMBO]->(subCombo:AcupointCombo)

RETURN collect(DISTINCT subCombo.name) AS sub_combos

}

RETURN

c.name AS combo_name, c.indications AS indications,

c.acupuncture_method_json AS acupuncture_method,

std_points, local_points, sub_combos

```

### 6.7 disease_compare_plans

Cypher

```

MATCH (d:Disease {name: $disease_name})-[:HAS_PLAN]->(p:TreatmentPlan)CALL {

WITH p

OPTIONAL MATCH (p)-[:MAIN_POINT]->(mc:AcupointCombo)

RETURN collect(DISTINCT mc.name) AS main_combos

}

CALL {

WITH p

OPTIONAL MATCH (p)-[:AUX_POINT]->(ac:AcupointCombo)

RETURN collect(DISTINCT ac.name) AS aux_combos

}

CALL {

WITH p

OPTIONAL MATCH (p)-[:MAIN_POINT]->(mp:Acupoint)

RETURN collect(DISTINCT mp.name) AS main_points

}

CALL {

WITH p

OPTIONAL MATCH (p)-[:AUX_POINT]->(ap:Acupoint)

RETURN collect(DISTINCT ap.name) AS aux_points

}

RETURN

d.name AS disease, p.plan_id AS plan_id,

p.method_text AS method_text, p.course_text AS course_text,

p.effect_text AS effect_text, p.effect_level AS effect_level,

p.has_electroacupuncture AS has_electroacupuncture,

p.has_moxibustion AS has_moxibustion, p.has_drug AS has_drug,

main_combos, aux_combos, main_points, aux_points

ORDER BY p.effect_level DESC, p.plan_id

LIMIT $limit

```

### 6.8 combo_detail

cypher

```

MATCH (c:AcupointCombo {name: $combo_name})CALL {

WITH c

OPTIONAL MATCH (c)-[r1:HAS_POINT]->(stdPoint:Acupoint)

RETURN collect(DISTINCT {name: stdPoint.name, needle_method: r1.needle_method}) AS std_points

}

CALL {

WITH c

OPTIONAL MATCH (c)-[r2:HAS_LOCAL_POINT]->(localPoint:ComboLocalPoint)

RETURN collect(DISTINCT {name: localPoint.name, needle_method: r2.needle_method}) AS local_points

}

CALL {

WITH c

OPTIONAL MATCH (c)-[:HAS_COMBO]->(subCombo:AcupointCombo)

RETURN collect(DISTINCT subCombo.name) AS sub_combos

}

CALL {

WITH c

OPTIONAL MATCH (d:Disease)-[:HAS_PLAN]->(p:TreatmentPlan)

WHERE (p)-[:MAIN_POINT]->(c) OR (p)-[:AUX_POINT]->(c)

RETURN collect(DISTINCT d.name) AS related_diseases, count(DISTINCT p) AS plan_count

}

RETURN

c.name AS combo_name, c.indications AS indications,

c.acupuncture_method_json AS acupuncture_method,

std_points, local_points, sub_combos,

related_diseases, plan_count

```

### 6.9 multi_disease_common

This query type executes a per-disease query and then computes set intersections in Python. The per-disease Cypher is:

cypher

```

MATCH (d:Disease {name: $disease_name})-[:HAS_PLAN]->(p:TreatmentPlan)OPTIONAL MATCH (p)-[:MAIN_POINT]->(mc:AcupointCombo)

OPTIONAL MATCH (p)-[:AUX_POINT]->(ac:AcupointCombo)

OPTIONAL MATCH (p)-[:MAIN_POINT]->(mp:Acupoint)

OPTIONAL MATCH (p)-[:AUX_POINT]->(ap:Acupoint)

WITH d.name AS disease,

collect(DISTINCT mc.name) + collect(DISTINCT ac.name) AS all_combos,

collect(DISTINCT mp.name) + collect(DISTINCT ap.name) AS all_points

RETURN disease,

[x IN all_combos WHERE x IS NOT NULL] AS all_combos,

[x IN all_points WHERE x IS NOT NULL] AS all_points

```

After executing this query for each disease, the system computes the intersection of combo sets and point sets across all queried diseases in Python, producing a result dictionary containing the disease list, common combinations, common acupoints, and per-disease detail.

## 7. Fallback Search Cypher Templates

When the primary query returns no results, the system attempts progressively broader substring matches.

### 7.1 Disease Fallback

Used when the query type involves diseases (disease_to_plans, disease_to_point_summary, disease_combo_to_effect, disease_compare_plans):

cypher

```

MATCH (d:Disease)-[:HAS_PLAN]->(p:TreatmentPlan)WHERE d.name CONTAINS $keyword

OPTIONAL MATCH (p)-[:MAIN_POINT]->(mc:AcupointCombo)

RETURN d.name AS disease, p.plan_id AS plan_id,

p.effect_text AS effect_text, p.effect_level AS effect_level,

collect(DISTINCT mc.name) AS main_combos

ORDER BY p.plan_id LIMIT $limit

The system tries the full disease name first, then the first three characters, then the first two characters, stopping at the first successful result.

```

### 7.2 Combination Fallback

Used when the query type involves combinations (combo_to_diseases, combo_to_points, combo_detail):

cypher

```

MATCH (c:AcupointCombo) WHERE c.name CONTAINS $keywordOPTIONAL MATCH (d:Disease)-[:HAS_PLAN]->(p:TreatmentPlan)

WHERE (p)-[:MAIN_POINT]->(c) OR (p)-[:AUX_POINT]->(c)

RETURN c.name AS combo_name, c.indications AS indications,

collect(DISTINCT d.name) AS diseases

LIMIT $limit

```

### 7.3 Acupoint Fallback

Used when the query type is point_to_combos:

Cypher

```

MATCH (a:Acupoint) WHERE a.name CONTAINS $keywordOPTIONAL MATCH (combo:AcupointCombo)-[:HAS_POINT]->(a)

RETURN a.name AS point_name, collect(DISTINCT combo.name) AS combos

LIMIT $limit

```

## 8. Error Handling and Fault Tolerance

The system implements fault tolerance at multiple levels. LLM parsing failures trigger a fallback to keyword-based heuristic rules. LLM entity alignment failures return an empty mapping without blocking the main pipeline. Empty Cypher query results activate the three-stage fuzzy fallback search described in Section 7. LLM answer generation failures produce degraded text responses that differ based on whether graph records were successfully retrieved: when records exist, the user is advised to consult the raw records directly; when no records exist, the user is directed to authoritative textbooks and clinical guidelines. All exceptions are logged via the Python logging module, and the API layer returns HTTP 500 with error details for unhandled exceptions.
