# Supplementary figures and images for: Construction and evaluation of the knowledge graph and large model question-answering system for Jin San Zhen therapy: a tool study for primary care and general practice
Source: Front Med (Lausanne). 2026 Apr 9;13:1755583. doi: 10.3389/fmed.2026.1755583 (PMC13103959; doi:10.3389/fmed.2026.1755583)

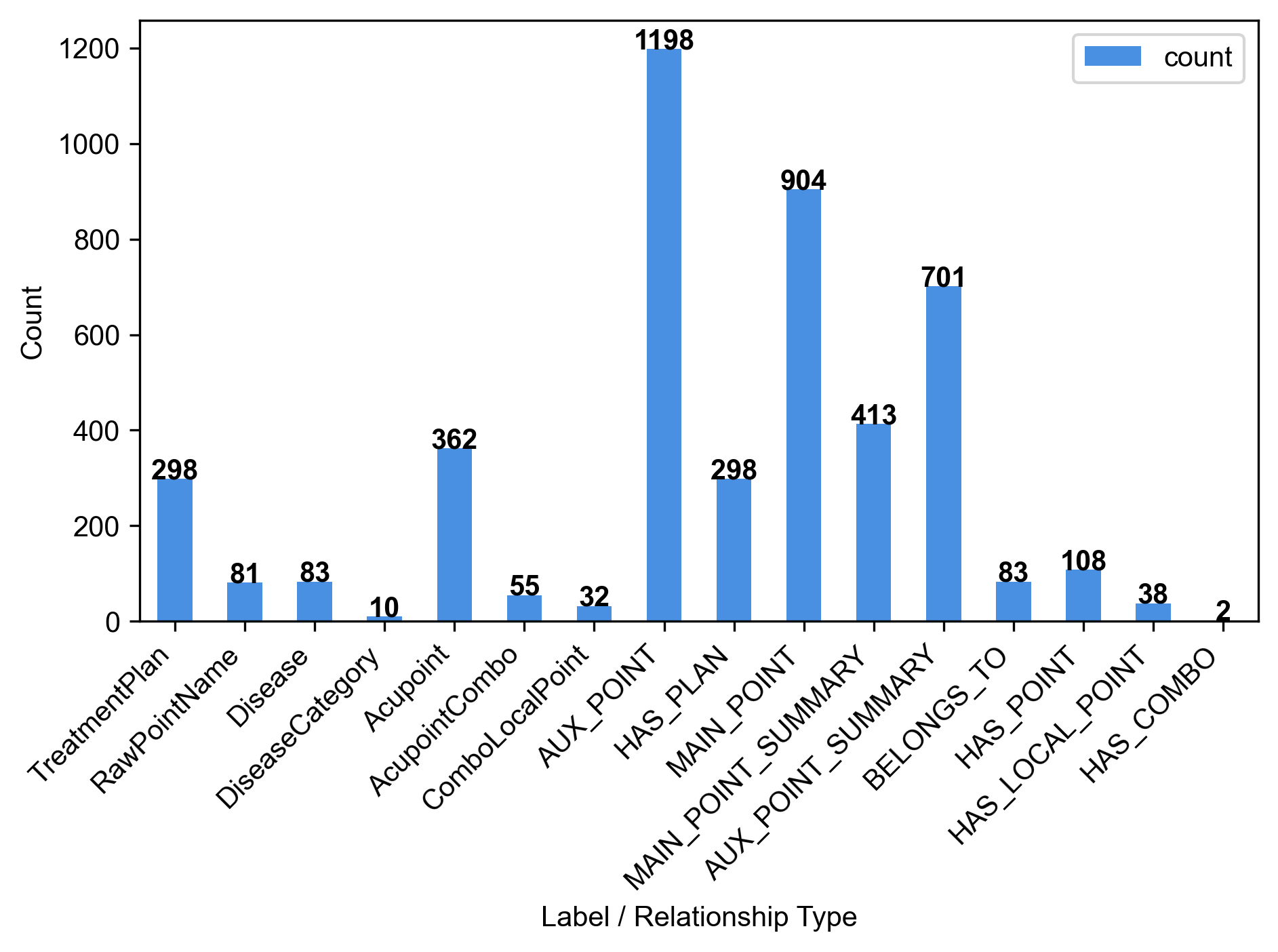

Supplement: Supplementary file 2 [file Image_1.png]

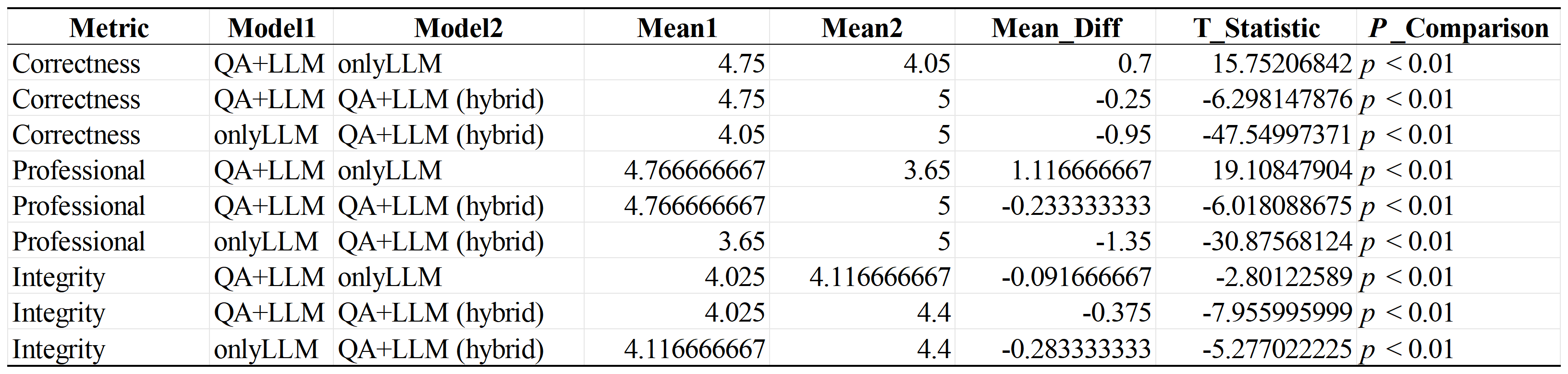

Supplement: Supplementary file 3 [file Image_2.png]
